# Supplementary material for: Antenatal Sildenafil for Congenital Diaphragmatic Hernia: A Systematic Review and Bayesian Meta-Analysis of Preclinical Studies
Source: Biomedicines. 2025 Sep 16;13(9):2274. doi: 10.3390/biomedicines13092274 (PMC12467270; doi:10.3390/biomedicines13092274)
Supplement: Supplementary file 1 [file biomedicines-13-02274-s001.zip › biomedicines-3831908-supplementary.pdf]

Supplementary Online Content

Antenatal sildenafil for Congenital Diaphragmatic Hernia: A Systematic Review and Bayesian Meta-analysis of Preclinical Studies

Tamara M. Hundscheid, Ilaria Amodeo, Giacomo Cavallaro, Carlijn R Hooijmans, František Bartoš, and Eduardo Villamor

Search strategy

PubMed

((“Phosphodiesterase 5 Inhibitors” [Mesh] OR “Phosphodiesterase 5” [tiab] OR “Phosphodiesterase type 5” [tiab] OR “Phosphodiesterase V” [tiab] OR “PDE type 5” [tiab] OR “PDE-5” [tiab] OR “PDE5” [tiab] OR “PDE type V” [tiab] OR “PDE V” [tiab] OR “PDE-V” [tiab] OR “Phosphodiesterase type 5” [tiab] OR “PDE5” [tiab] OR “PDE-5” [tiab] OR “PDE 5” [tiab] OR “Sildenafil citrate” [Mesh] OR “NCX-911” [tiab] OR “NCX 911” [tiab] OR “NCX911” [tiab] OR “Revatio” [tiab] OR “sildenafil” [tiab] OR “UK 92480-10” [tiab] OR “UK 92480 10” [tiab] OR “UK 9248010” [tiab] OR “Viagra” [tiab] OR “acetildenafil” [tiab] OR “tadalafil” [Mesh] OR “tadalafil” [tiab] OR “IC351” [tiab] OR “IC-351” [tiab] OR “IC 351” [tiab] OR “Cialis” OR “vardenafil dihydrochloride” [Mesh] OR “Vardenafil” [tiab] OR “Levitra” [tiab] OR “avanafil” [tiab] OR “adcirca” [tiab] OR “staxyn” [tiab] OR “vivanza” [tiab] OR “stendra” [tiab] OR “spedra” [tiab] OR “lodenafil” [tiab] OR “helleva” [tiab] OR “mirodenafil” [tiab] OR “mvix” [tiab] OR “udenafil” [tiab] OR “zydena” [tiab] OR “zaprinast” [tiab] OR “benzamidenafil” [tiab])

AND

(“Hernias, Diaphragmatic, Congenital”[Mesh] OR “congenital diaphragmatic hernia” [tiab] OR “congenital diaphragmatic hernias” [tiab] OR “Congenital Diaphragmatic Defect” [tiab] OR “Congenital Diaphragmatic Defects” [tiab] OR “Agenesis of Hemidiaphragm” [tiab] OR “Hemidiaphragm Ageneses” [tiab] OR “Hemidiaphragm Agenesis” [tiab] OR “Unilateral Agenesis of Diaphragm” [tiab] OR “Diaphragm Unilateral Ageneses” [tiab] OR “Diaphragm Unilateral Agenesis” [tiab] OR “Bochdalek Hernia” [tiab] OR “Bochdalek Hernias” [tiab] OR “Morgagni Hernia” [tiab] OR “Morgagni Hernias” [tiab] OR “Morgagni's Hernia” [tiab] OR “Morgagni's Hernias” [tiab] OR “Morgagnis Hernia” [tiab] OR “Morgagnis Hernias” [tiab] OR “congenital hernia diaphragmatica” [tiab] OR “diaphragm congenital defect” [tiab] OR “diaphragm congenital hernia” [tiab] OR “hernia diaphragmatica congenital” [tiab] OR “CDH” [tiab] OR “Diaphragmatic Experimental Hernia” [tiab] OR “nitrofen” [tiab] OR “diaphragmatic hernia” [tiab] OR ((congenital) AND (("Hernia, Diaphragmatic"[Mesh:noexp]) OR diaphragmatic hernia*)

AND

("animal experimentation"[MeSH Terms] OR "models, animal"[MeSH Terms] OR "invertebrates"[MeSH Terms] OR "Animals"[Mesh:noexp] OR "animal population groups"[MeSH Terms] OR "chordata"[MeSH Terms:noexp] OR "chordata, nonvertebrate"[MeSH Terms] OR "vertebrates"[MeSH Terms:noexp] OR "amphibians"[MeSH Terms] OR "birds"[MeSH Terms] OR "fishes"[MeSH Terms] OR "reptiles"[MeSH Terms] OR "mammals"[MeSH Terms:noexp] OR "primates"[MeSH Terms:noexp] OR "artiodactyla"[MeSH Terms] OR "carnivora"[MeSH Terms] OR "cetacea"[MeSH Terms] OR "chiroptera"[MeSH Terms] OR "elephants"[MeSH Terms] OR "hyraxes"[MeSH Terms] OR "insectivora"[MeSH Terms] OR "lagomorpha"[MeSH Terms] OR "marsupialia"[MeSH Terms] OR "monotremata"[MeSH Terms] OR "perissodactyla"[MeSH Terms] OR "rodentia"[MeSH Terms] OR "scandentia"[MeSH Terms] OR "sirenia"[MeSH Terms] OR "xenarthra"[MeSH Terms] OR "haplorhini"[MeSH Terms:noexp] OR "strepsirhini"[MeSH Terms] OR "platyrrhini"[MeSH Terms] OR "tarsii"[MeSH Terms] OR "catarrhini"[MeSH Terms:noexp] OR "cercopithecidae"[MeSH Terms] OR "hylobatidae"[MeSH Terms] OR "hominidae"[MeSH Terms:noexp] OR "gorilla gorilla"[MeSH Terms] OR "pan paniscus"[MeSH Terms] OR "pan troglodytes"[MeSH Terms] OR "pongo pygmaeus"[MeSH Terms]) OR ((animals[tiab] OR animal[tiab] OR mice[Tiab] OR mus[Tiab] OR mouse[Tiab] OR murine[Tiab] OR woodmouse[tiab] OR rats[Tiab] OR

rat[Tiab] OR murinae[Tiab] OR muridae[Tiab] OR cottonrat[tiab] OR cottonrats[tiab] OR hamster[tiab] OR hamsters[tiab] OR cricetinae[tiab] OR rodentia[Tiab] OR rodent[Tiab] OR rodents[Tiab] OR pigs[Tiab] OR pig[Tiab] OR swine[tiab] OR swines[tiab] OR piglets[tiab] OR piglet[tiab] OR boar[tiab] OR boars[tiab] OR "sus scrofa"[tiab] OR ferrets[tiab] OR ferret[tiab] OR polecat[tiab] OR polecats[tiab] OR "mustela putorius"[tiab] OR "guinea pigs"[Tiab] OR "guinea pig"[Tiab] OR cavia[Tiab] OR callithrix[Tiab] OR marmoset[Tiab] OR marmosets[Tiab] OR cebuella[Tiab] OR hapale[Tiab] OR octodon[Tiab] OR chinchilla[Tiab] OR chinchillas[Tiab] OR gerbillinae[Tiab] OR gerbil[Tiab] OR gerbils[Tiab] OR jird[Tiab] OR jirds[Tiab] OR merione[Tiab] OR meriones[Tiab] OR rabbits[Tiab] OR rabbit[Tiab] OR hares[Tiab] OR hare[Tiab] OR diptera[Tiab] OR flies[Tiab] OR fly[Tiab] OR dipteral[Tiab] OR drosphila[Tiab] OR drosophilidae[Tiab] OR cats[Tiab] OR cat[Tiab] OR carus[Tiab] OR felis[Tiab] OR nematoda[Tiab] OR nematode[Tiab] OR nematoda[Tiab] OR nematode[Tiab] OR nematodes[Tiab] OR sipunculida[Tiab] OR dogs[Tiab] OR dog[Tiab] OR canine[Tiab] OR canines[Tiab] OR canis[Tiab] OR sheep[Tiab] OR sheeps[Tiab] OR mouflon[Tiab] OR mouflons[Tiab] OR ovis[Tiab] OR goats[Tiab] OR goat[Tiab] OR capra[Tiab] OR capras[Tiab] OR rupicapra[Tiab] OR chamois[Tiab] OR haplorhini[Tiab] OR monkey[Tiab] OR monkeys[Tiab] OR anthropoidea[Tiab] OR anthropoids[Tiab] OR saguinus[Tiab] OR tamarin[Tiab] OR tamarins[Tiab] OR leontopithecus[Tiab] OR hominidae[Tiab] OR ape[Tiab] OR apes[Tiab] OR pan[Tiab] OR paniscus[Tiab] OR "pan paniscus"[Tiab] OR bonobo[Tiab] OR bonobos[Tiab] OR troglodytes[Tiab] OR "pan troglodytes"[Tiab] OR gibbon[Tiab] OR gibbons[Tiab] OR siamang[Tiab] OR siamangs[Tiab] OR nomascus[Tiab] OR symphalangus[Tiab] OR chimpanzee[Tiab] OR chimpanzees[Tiab] OR prosimians[Tiab] OR "bush baby"[Tiab] OR prosimian[Tiab] OR bush babies[Tiab] OR galagos[Tiab] OR galago[Tiab] OR pongidae[Tiab] OR gorilla[Tiab] OR gorillas[Tiab] OR pongo[Tiab] OR pygmaeus[Tiab] OR "pongo pygmaeus"[Tiab] OR orangutans[Tiab] OR pygmaeus[Tiab] OR lemur[Tiab] OR lemurs[Tiab] OR lemuridae[Tiab] OR horse[Tiab] OR horses[Tiab] OR pongo[Tiab] OR equus[Tiab] OR cow[Tiab] OR calf[Tiab] OR bull[Tiab] OR chicken[Tiab] OR chickens[Tiab] OR gallus[Tiab] OR quail[Tiab] OR bird[Tiab] OR birds[Tiab] OR quails[Tiab] OR poultry[Tiab] OR poultries[Tiab] OR fowl[Tiab] OR fowls[Tiab] OR reptile[Tiab] OR reptilia[Tiab] OR reptiles[Tiab] OR snakes[Tiab] OR snake[Tiab] OR lizard[Tiab] OR lizards[Tiab] OR alligator[Tiab] OR alligators[Tiab] OR crocodile[Tiab] OR crocodiles[Tiab] OR turtle[Tiab] OR turtles[Tiab] OR amphibian[Tiab] OR amphibians[Tiab] OR amphibia[Tiab] OR frog[Tiab] OR frogs[Tiab] OR bombina[Tiab] OR salientia[Tiab] OR toad[Tiab] OR toads[Tiab] OR "epidalea calamita"[Tiab] OR salamander[Tiab] OR salamanders[Tiab] OR eel[Tiab] OR eels[Tiab] OR fish[Tiab] OR fishes[Tiab] OR pisces[Tiab] OR catfish[Tiab] OR catfishes[Tiab] OR siluriformes[Tiab] OR arius[Tiab] OR heteropneustes[Tiab] OR sheatfish[Tiab] OR perch[Tiab] OR perches[Tiab] OR percidae[Tiab] OR perca[Tiab] OR trout[Tiab] OR trouts[Tiab] OR char[Tiab] OR chars[Tiab] OR salvelinus[Tiab] OR "fathead minnow"[Tiab] OR minnow[Tiab] OR cyprinidae[Tiab] OR carps[Tiab] OR carp[Tiab] OR zebrafish[Tiab] OR zebrafishes[Tiab] OR goldfish[Tiab] OR goldfishes[Tiab] OR guppy[Tiab] OR guppies[Tiab] OR chub[Tiab] OR chubs[Tiab] OR tinca[Tiab] OR barbels[Tiab] OR barbus[Tiab] OR pimephales[Tiab] OR promelas[Tiab] OR "poecilia reticulata"[Tiab] OR mullet[Tiab] OR mullets[Tiab] OR seahorse[Tiab] OR seahorses[Tiab] OR mugil curema[Tiab] OR atlantic cod[Tiab] OR shark[Tiab] OR sharks[Tiab] OR catshark[Tiab] OR anguilla[Tiab] OR salmonid[Tiab] OR salmonids[Tiab] OR whitefish[Tiab] OR whitefishes[Tiab] OR salmon[Tiab] OR salmons[Tiab] OR sole[Tiab] OR solea[Tiab] OR "sea lamprey"[Tiab] OR lamprey[Tiab] OR lampreys[Tiab] OR pumpkinseed[Tiab] OR sunfish[Tiab] OR sunfishes[Tiab] OR tilapia[Tiab] OR tilapias[Tiab] OR turbot[Tiab] OR turbots[Tiab] OR flatfish[Tiab] OR flatfishes[Tiab] OR sciuridae[Tiab] OR squirrel[Tiab] OR squirrels[Tiab] OR chipmunk[Tiab] OR chipmunks[Tiab] OR suslik[Tiab] OR susliks[Tiab] OR vole[Tiab] OR voles[Tiab] OR lemming[Tiab] OR lemmings[Tiab] OR muskrat[Tiab] OR muskrats[Tiab] OR lemmus[Tiab] OR otter[Tiab] OR otters[Tiab] OR marten[Tiab] OR martens[Tiab] OR martes[Tiab] OR weasel[Tiab] OR badger[Tiab] OR badgers[Tiab] OR ermine[Tiab] OR mink[Tiab] OR minks[Tiab] OR sable[Tiab] OR sables[Tiab] OR gulo[Tiab] OR gulos[Tiab] OR wolverine[Tiab] OR wolverines[Tiab] OR minks[Tiab] OR mustela[Tiab] OR llama[Tiab] OR llamas[Tiab] OR alpaca[Tiab] OR alpacas[Tiab] OR camelid[Tiab] OR camelids[Tiab] OR guanaco[Tiab] OR guanacos[Tiab] OR chiroptera[Tiab] OR chiropteras[Tiab] OR bat[Tiab] OR bats[Tiab] OR fox[Tiab] OR foxes[Tiab] OR iguana[Tiab] OR iguanas[Tiab] OR xenopus laevis[Tiab] OR parakeet[Tiab] OR parakeets[Tiab] OR parrot[Tiab] OR parrots[Tiab] OR donkey[Tiab] OR donkeys[Tiab] OR mule[Tiab] OR mules[Tiab] OR zebra[Tiab] OR zebras[Tiab] OR shrew[Tiab] OR shrews[Tiab] OR bison[Tiab] OR bisons[Tiab] OR buffalo[Tiab] OR buffaloes[Tiab] OR deer[Tiab] OR deers[Tiab] OR bear[Tiab] OR bears[Tiab] OR panda[Tiab] OR pandas[Tiab] OR "wild hog"[Tiab] OR "wild boar"[Tiab]

OR fitchew[Tiab] OR fitch[Tiab] OR beaver[Tiab] OR beavers[Tiab] OR jerboa[Tiab] OR jerboas[Tiab] OR capybara[Tiab] OR capybaras[Tiab]) NOT medline[subset])))

Embase

((exp Phosphodiesterase V inhibitor/ OR exp Sildenafil/ OR exp tadalafil/ OR exp vardenafil/ OR exp avanafil/ OR (Phosphodiesterase 5 OR Phosphodiesterase type 5 OR Phosphodiesterase V OR Phosphodiesterase type V OR PDE type 5 OR PDE type V OR PDE5 OR PDE 5 OR PDE V OR PDE-V inhibitor OR PDE-5 OR PDE-5 OR PDE 5 OR sildenafil OR NCX-911 OR NCX 911 OR NCX911 OR revatio OR UK 92480-10 OR UK 92480 10 OR UK 9248010 OR UK-92,480-10 OR UK 92,480 10 OR Viagra OR acetildenafil OR tadalafil OR IC351 OR IC-351 OR IC 351 OR Cialis OR Vardenafil OR Levitra OR avanafil OR adcirca OR staxyn OR vivanza OR stendra OR spedra OR lodenafil OR helleva OR mirodenafil OR mvix OR udenafil OR zydena OR zaprinast OR benzamidenafil).ti,ab.)

AND

(exp Congenital diaphragm hernia/ OR (Congenital Diaphragmatic Defect OR Congenital Diaphragmatic Defects OR Defect, Congenital Diaphragmatic OR Defects, Congenital Diaphragmatic OR Diaphragmatic Defect, Congenital OR Diaphragmatic Defects, Congenital OR Agenesis of Hemidiaphragm OR Hemidiaphragm Ageneses OR Hemidiaphragm Agenesis OR Unilateral Agenesis of Diaphragm OR Diaphragm Unilateral Ageneses OR Diaphragm Unilateral Agenesis OR Congenital Diaphragmatic Hernia OR Diaphragmatic Hernia, Congenital OR Diaphragmatic Hernias, Congenital OR Hernia, Congenital Diaphragmatic OR Hernias, Congenital Diaphragmatic OR congenital diaphragmatic hernias OR congenital diaphragmatic hernia OR Bochdalek Hernias OR Bochdalek Hernia OR Hernia, Bochdalek OR Hernias, Bochdalek OR Morgagni Hernias OR Morgagni Hernia OR Morgagnis Hernias OR Morgagnis Hernia OR congenital hernia diaphragmatica OR diaphragm congenital defect OR diaphragm congenital hernia OR diaphragm hernia, congenital OR hernia diaphragmatica congenital OR hernias, diaphragmatic, congenital OR CDH OR Diaphragmatic Experimental Hernia OR diaphragmatic hernia OR diaphragmatic hernias OR hernias, diaphragmatic OR nitrofen).ti,ab. OR ((congenital.ti,ab.) AND (exp diaphragm hernia/ OR diaphragmatic hernia*.ti,ab.)))

AND

(exp animal experiment/ or exp animal model/ or exp experimental animal/ or exp transgenic animal/ or exp male animal/ or exp female animal/ or exp juvenile animal/ OR animal/ OR chordata/ OR vertebrate/ OR tetrapod/ OR exp fish/ OR amniote/ OR exp amphibia/ OR mammal/ OR exp reptile/ OR exp sauropsid/ OR therian/OR exp monotremate/ OR placental mammals/ OR exp marsupial/ OR Euarchontoglires/ OR exp Afrotheria/ OR exp Boreoeutheria/ OR exp Laurasiatheria/ OR exp Xenarthra/ OR primate/ OR exp Dermoptera/ OR exp Glires/ OR exp Scandentia/ OR Haplorhini/ OR exp prosimian/ OR simian/ OR exp tarsiiform/ OR Catarrhini/ OR exp Platyrrhini/ OR ape/ OR exp Cercopithecidae/ OR hominid/ OR exp hylobatidae/ OR exp chimpanzee/ OR exp gorilla/ OR exp orang utan/ OR (animal OR animals OR pisces OR fish OR fishes OR catfish OR catfishes OR sheatfish OR silurus OR arius OR heteropneustes OR clarias OR gariepinus OR fathead minnow OR fathead minnows OR pimephales OR promelas OR cichlidae OR trout OR trouts OR char OR chars OR salvelinus OR salmo OR oncorhynchus OR guppy OR guppies OR millionfish OR poecilia OR goldfish OR goldfishes OR carassius OR auratus OR mullet OR mullets OR mugil OR curema OR shark OR sharks OR cod OR cods OR gadus OR morhua OR carp OR carps OR cyprinus OR carpio OR killifish OR eel OR eels OR anguilla OR zander OR sander OR lucioperca OR stizostedion OR turbot OR turbots OR psetta OR flatfish OR flatfishes OR plaice OR pleuronectes OR platessa OR tilapia OR tilapias OR oreochromis OR sarotherodon OR common sole OR dover sole OR solea OR zebrafish OR zebrafishes OR danio OR rerio OR seabass OR dicentrarchus OR labrax OR morone OR lamprey OR lampreys OR petromyzon OR pumpkinseed OR pumpkinseeds OR lepomis OR gibbosus OR herring OR clupea OR harengus OR amphibia OR amphibian OR amphibians OR anura OR salientia OR frog OR frogs OR rana OR toad OR toads OR bufo OR xenopus OR laevis OR bombina OR epidalea OR calamita OR salamander OR salamanders OR newt OR newts OR triturus OR reptilia OR reptile OR reptiles OR bearded dragon OR pogona OR vitticeps OR iguana OR iguanas OR lizard OR lizards OR anguis fragilis OR turtle OR turtles OR snakes OR snake OR aves OR bird OR birds OR quail OR quails OR coturnix OR bobwhite OR colinus OR virginianus OR poultry OR poultries OR fowl OR fowls OR chicken OR chickens

OR gallus OR zebra finch OR taeniopygia OR guttata OR canary OR canaries OR serinus OR canaria OR parakeet OR parakeets OR grasskeet OR parrot OR parrots OR psittacine OR psittacines OR shelduck OR tadorna OR goose OR geese OR branta OR leucopsis OR woodlark OR lullula OR flycatcher OR ficedula OR hypoleuca OR dove OR doves OR geopelia OR cuneata OR duck OR ducks OR greylag OR graylag OR anser OR harrier OR circus pygargus OR red knot OR great knot OR calidris OR canutus OR godwit OR limosa OR lapponica OR meleagris OR gallopavo OR jackdaw OR corvus OR monedula OR ruff OR philomachus OR pugnax OR lapwing OR peewit OR plover OR vanellus OR swan OR cygnus OR columbianus OR bewickii OR gull OR chroicocephalus OR ridibundus OR albifrons OR great tit OR parus OR aythya OR fuligula OR streptopelia OR risoria OR spoonbill OR platalea OR leucorodia OR blackbird OR turdus OR merula OR blue tit OR cyanistes OR pigeon OR pigeons OR columba OR pintail OR anas OR starling OR sturnus OR owl OR athene noctua OR pochard OR ferina OR cockatiel OR nymphicus OR hollandicus OR skylark OR alauda OR tern OR sterna OR teal OR crecca OR oystercatcher OR haematopus OR ostralegus OR shrew OR shrews OR sorex OR araneus OR crocidura OR russula OR european mole OR talpa OR chiroptera OR bat OR bats OR eptesicus OR serotinus OR myotis OR dasycneme OR daubentonii OR pipistrelle OR pipistrellus OR cat OR cats OR felis OR catus OR feline OR dog OR dogs OR canis OR canine OR canines OR otter OR otters OR lutra OR badger OR badgers OR meles OR fitchew OR fitch OR foumart or foulmart OR ferrets OR ferret OR polecat OR polecats OR mustela OR putorius OR weasel OR weasels OR fox OR foxes OR vulpes OR common seal OR phoca OR vitulina OR grey seal OR halichoerus OR horse OR horses OR equus OR equine OR equidae OR donkey OR donkeys OR mule OR mules OR pig OR pigs OR swine OR swines OR hog OR hogs OR boar OR boars OR porcine OR piglet OR piglets OR sus OR scrofa OR llama OR llamas OR lama OR glama OR deer OR deers OR cervus OR elaphus OR cow OR cows OR bos taurus OR bos indicus OR bovine OR bull OR bulls OR cattle OR bison OR bisons OR sheep OR sheeps OR ovis aries OR ovine OR lamb OR lambs OR mouflon OR mouflons OR goat OR goats OR capra OR caprine OR chamois OR rupicapra OR leporidae OR lagomorpha OR lagomorph OR rabbit OR rabbits OR oryctolagus OR cuniculus OR laprine OR hares OR lepus OR rodentia OR rodent OR rodents OR murinae OR mouse OR mice OR mus OR musculus OR murine OR woodmouse OR apodemus OR rat OR rats OR rattus OR norvegicus OR guinea pig OR guinea pigs OR cavia OR porcellus OR hamster OR hamsters OR mesocricetus OR cricetulus OR cricetus OR gerbil OR gerbils OR jird OR jirds OR meriones OR unguiculatus OR jerboa OR jerboas OR jaculus OR chinchilla OR chinchillas OR beaver OR beavers OR castor fiber OR castor canadensis OR sciuridae OR squirrel OR squirrels OR sciurus OR chipmunk OR chipmunks OR marmot OR marmots OR marmota OR suslik OR susliks OR spermophilus OR cynomys OR cottonrat OR cottonrats OR sigmodon OR vole OR voles OR microtus OR myodes OR glareolus OR primate OR primates OR prosimian OR prosimians OR lemur OR lemurs OR lemuridae OR loris OR bush baby OR bush babies OR bushbaby OR bushbabies OR galago OR galagos OR anthropoidea OR anthropoids OR simian OR simians OR monkey OR monkeys OR marmoset OR marmosets OR callithrix OR cebuella OR tamarin OR tamarins OR saguinus OR leontopithecus OR squirrel monkey OR squirrel monkeys OR saimiri OR night monkey OR night monkeys OR owl monkey OR owl monkeys OR douroucoulis OR aotus OR spider monkey OR spider monkeys OR ateles OR baboon OR baboons OR papio OR rhesus monkey OR macaque OR macaca OR mulatta OR cynomolgus OR fascicularis OR green monkey OR green monkeys OR chlorocebus OR vervet OR vervets OR pygerythrus OR hominoidea OR ape OR apes OR hylobatidae OR gibbon OR gibbons OR siamang OR siamangs OR nomascus OR symphalangus OR hominidae OR orangutan OR orangutans OR pongo OR chimpanzee OR chimpanzees OR pan troglodytes OR bonobo OR bonobos OR pan paniscus OR gorilla OR gorillas OR troglodytes).ti,ab.))

Supplementary Table 1. Frequentist random-effects meta-analysis of fetal survival, lung morphometry and function.

Outcome	Subgroup	k	Effect size	95% CI		P-value	Heterogeneity		
				Lower limit	Upper limit		Q-value	P-value	I^2(%)
Fetal survival	All	7	1.32[a]	0.78	2.25	0.305	12.60	0.050	52.4
LBWR	All	16	0.49[b]	-0.01	0.93	0.051	94.26	<0.001	84.1
	Nitrofen	13	0.45[b]	-0.05	0.94	0.076	83.60	<0.001	85.6
Distal airway complexity (MTBD)	All	3	-0.93[b]	-1.48	-0.38	<0.001	1.76	0.415	0.0
Mean saccular airspace diameter (D2 score)	All	2	1.68[b]	1.14	2.21	<0.001	0.01	0.906	0.0
Mean linear intercept	All	4	-0.21[b]	-1.40	0.99	0.731	26.64	<0.001	87.3
Radial alveolar count	All	6	1.09[b]	0.57	1.61	<0.001	7.68	0.175	34.9
Interalveolar septa thickness	All	4	-1.36[b]	-1.86	-0.86	<0.001	3.63	0.304	17.3
Total lung capacity	All	2	1.51[b]	0.71	2.31	<0.001	0.00	0.973	0.0
Compliance	All	2	1.33[b]	0.55	2.11	<0.001	0.18	0.675	0.0
Elastance	All	2	-0.99[b]	-1.73	-0.24	0.009	0.79	0.373	0.0

CI: confidence interval credible interval; K: number of studies; LBWR: Lung-to-body weight ratio. MTBD: mean terminal bronchiole density

[a]Odds ratio; [b]Hedges' *g*.

Supplementary Table 2. Frequentist random-effects meta-analysis of pulmonary vascular morphometry, heart morphometry and function.

Outcome	k	Hedges' g	95% CI		p-value	Heterogeneity		
			Lower limit	Upper limit		Q-value	P-value	I^2(%)
Medial wall thickness	12	-1.64	-2.21	-1.06	<0.001	84.02	<0.001	86.9
	10	-1.44	-2.03	-0.84	<0.001	73.68	<0.001	87.8
Proportionate medial thickness	3	-3.81	-5.89	-1.73	<0.001	7.43	0.024	73.1
Proportionate adventitial thickness	2	-0.91	-1.94	0.11	0.081	1.83	0.176	45.3
Vascular volume	2	0.09	-0.95	1.13	0.867	1.54	0.214	35.2
RV/LV + S	4	-0.53	-1.22	0.15	0.128	3.73	0.293	19.5
RV wall thickness	2	-4.41	-7.67	-1.15	0.008	3.72	<0.001	73.1
Doppler AT/ET	2	1.69	0.87	2.52	<0.001	0.08	0.778	0.0

AT/ET: Acceleration/Ejection Time ratio; CI: confidence interval; K: number of studies; RV/LV + S: right ventricle to left ventricle plus septum ratio.
